# Evaluation of *Escherichia coli* isolates from healthy chickens to determine their potential risk to poultry and human health

**DOI:** 10.1371/journal.pone.0180599

**Published:** 2017-07-03

**Authors:** Zachary R. Stromberg, James R. Johnson, John M. Fairbrother, Jacquelyn Kilbourne, Angelica Van Goor, Roy Curtiss, Melha Mellata

**Affiliations:** 1 Department of Food Science and Human Nutrition, Iowa State University, Ames, Iowa, United States of America; 2 Veterans Affairs Medical Center and University of Minnesota, Minneapolis, Minnesota, United States of America; 3 OIE Reference Laboratory for *Escherichia coli*, Faculty of Veterinary Medicine, Université de Montréal, St-Hyacinthe, Québec, Canada; 4 The Biodesign Institute, Arizona State University, Tempe, Arizona, United States of America; 5 School of Life Sciences, Arizona State University, Tempe, Arizona, United States of America; The Pennsylvania State University, UNITED STATES

## Abstract

Extraintestinal pathogenic *Escherichia coli* (ExPEC) strains are important pathogens that cause diverse diseases in humans and poultry. Some *E*. *coli* isolates from chicken feces contain ExPEC-associated virulence genes, so appear potentially pathogenic; they conceivably could be transmitted to humans through handling and/or consumption of contaminated meat. However, the actual extraintestinal virulence potential of chicken-source fecal *E*. *coli* is poorly understood. Here, we assessed whether fecal *E*. *coli* isolates from healthy production chickens could cause diseases in a chicken model of avian colibacillosis and three rodent models of ExPEC-associated human infections. From 304 *E*. *coli* isolates from chicken fecal samples, 175 *E*. *coli* isolates were screened by PCR for virulence genes associated with human-source ExPEC or avian pathogenic *E*. *coli* (APEC), an ExPEC subset that causes extraintestinal infections in poultry. Selected isolates genetically identified as ExPEC and non-ExPEC isolates were assessed *in vitro* for virulence-associated phenotypes, and *in vivo* for disease-causing ability in animal models of colibacillosis, sepsis, meningitis, and urinary tract infection. Among the study isolates, 13% (40/304) were identified as ExPEC; the majority of these were classified as APEC and uropathogenic *E*. *coli*, but none as neonatal meningitis *E*. *coli*. Multiple chicken-source fecal ExPEC isolates resembled avian and human clinical ExPEC isolates in causing one or more ExPEC-associated illnesses in experimental animal infection models. Additionally, some isolates that were classified as non-ExPEC were able to cause ExPEC-associated illnesses in animal models, and thus future studies are needed to elucidate their mechanisms of virulence. These findings show that *E*. *coli* isolates from chicken feces contain ExPEC-associated genes, exhibit ExPEC-associated *in vitro* phenotypes, and can cause ExPEC-associated infections in animal models, and thus may pose a health threat to poultry and consumers.

## Introduction

The primary and secondary habitats of *Escherichia coli* are the intestinal tract of warm-blooded animals and the environment, respectively. In poultry, as in humans, *E*. *coli* resides in the lower digestive tract, which it colonizes in the first 24 h after hatching [1] or birth [2]. Although many *E*. *coli* strains are harmless commensals, a subset have acquired the ability to cause intestinal or extraintestinal diseases. Extraintestinal pathogenic *E*. *coli* (ExPEC) strains cause diverse infections outside of the intestinal tract in humans and animals [3–5]. Based on the host and the site of infection, different ExPEC strains are subclassified as neonatal meningitis *E*. *coli* (NMEC), sepsis-associated *E*. *coli* (SEPEC), uropathogenic *E*. *coli* (UPEC), which cause newborn meningitis, sepsis, and urinary tract infections (UTI), respectively; and avian pathogenic *E*. *coli* (APEC), which mainly causes respiratory and systemic disease in poultry.

ExPEC infections are important to human health and are a major cause of economic loss to the poultry industry. In the United States, the costs associated with ExPEC infections in humans and poultry exceeds $4 billion per year [3, 6]. ExPEC strains can colonize the intestine, similar to non-pathogenic commensal *E*. *coli* [3, 6], but are equipped with virulence factors that allow them to cause disease in extraintestinal sites. In addition to the intestine, poultry houses serve as a reservoir for APEC [7], and this environment allows strains to persist for many months over successive flocks [8].

Epidemiological studies have documented the presence of ExPEC, as defined by molecular criteria, in the intestine of healthy poultry and in poultry meat, with some strains being genetically similar to those responsible for human infections [7, 9, 10]. Based on epidemiological analysis and molecular typing, it is suspected that food-producing animals are a source of bacteria capable of causing human ExPEC infections [11]. However, the frequency with which humans acquire ExPEC through consumption or handling of ExPEC-contaminated foods, become colonized intestinally, and subsequently develop infection at extraintestinal sites, is undefined [12].

Chicken-to-chicken ExPEC transmission, through pecking or inhalation of contaminated fecal dust could result in carcass condemnation and severe disease or death of poultry [3, 8]. In addition, ExPEC transmission among chickens may increase the presence of ExPEC colonized chickens, and thus increase the frequency of ExPEC transmission onto poultry products. Fecal contamination of poultry carcasses at slaughter, including from rupture of the digestive system during processing, is likely a major source of meat contamination with ExPEC [7, 10, 13]. Such organisms could be transmitted to humans through consumption of contaminated meat, cross-contamination of non-meat items during food preparation, hand-mouth contamination by the food preparer, or direct human-animal contact [11].

Improved understandings of the risk of chicken-source fecal *E*. *coli* are needed to guide the development of preventative measures to reduce infection in poultry and subsequent food contamination. Accordingly, this study's objectives were (i) to characterize *E*. *coli* isolates from chicken fecal samples both genetically and phenotypically for virulence-associated traits and (ii) to determine the virulence of selected isolates in animal models of chicken colibacillosis and human ExPEC diseases (sepsis, meningitis, and UTI).

## Materials and methods

### Human and animal ethics statement

With approval from the Arizona State University (ASU) Institutional Review Board (#1012005820) and the subjects' written informed consent, voided urine was collected from 2 male and 2 female healthy adult human volunteers. Animal infection experiments were performed in dedicated animal facilities in accordance with protocols approved by the ASU or Iowa State University (ISU) Institutional Animal Care and Use Committee (ASU Protocol number 1168R and ISU Protocol number 1-16-8159G). Appropriate procedures were used to reduce potential pain, distress, and discomfort. Animals were acclimated for 7 days before each experiment and received enrichment devices. Animals were housed in groups in order to promote social behavior. Humane endpoint criteria were set for all animals such that any moribund animal, animals exhibiting immobility (unable to feed or drink) or failure to groom (rodents only) were euthanized immediately according to the recommendations of the American Veterinary Medical Association 2013 Guidelines, and all remaining animals were euthanized at specific time points post-inoculation as described below. Animals exhibiting signs of illness but not meeting endpoint were not treated to maintain critical experimental data (e.g., bacterial loads), instead specific early endpoints were used as described below to minimize suffering.

### Bacterial strains and growth media

Bacterial strains were routinely grown at 37°C in Luria Bertani (LB) broth, on LB agar, or on MacConkey agar unless stated otherwise. Freezer stocks were maintained at -80°C in peptone-glycerol medium. Positive control *E*. *coli* strains for the following disease models included urosepsis isolate CFT073 [14] for sepsis and UTI, cystitis isolate UTI89 [15] for UTI, neonatal meningitis isolate RS218 [16] for meningitis, and avian-source χ7122 [17] and APEC-O2 [18] for avian colibacillosis. Negative control strains included *E*. *coli* K-12 MG1655 for sepsis, UTI, and colibacillosis, and laboratory *E*. *coli* strain DH5α for meningitis. For colicin production, *E*. *coli* K-12 χ6092 was used as a sensitive indicator [19].

Three-hundred and four fecal *E*. *coli* isolates were obtained from conventionally-raised commercial chickens. For this, fresh fecal samples from the pen floor were collected from 7 different broiler chicken farms in Quebec, Canada. Each farm housed 20,000 to 30,000 chickens aged from 35 to 50 days at the time of sampling From each farm, 5–15 pooled fecal samples were suspended 1/10 (weight/volume) in buffered peptone water and enriched overnight at 37°C. Boiled DNA extracts from these cultures were tested by PCR [20] for the presence of the virulence genes *tsh*, *papC*, *iucD*, and *cnf*, which are associated with *E*. *coli* causing extraintestinal infections in one or more production animal species or humans [21–25], thereby permitting a rapid and inexpensive initial screen for a wide spectrum of possible ExPEC strains.

For screen-positive samples, enriched broths were streaked onto MacConkey agar. Three to 10 lactose-positive (i.e., presumptive *E*. *coli*) colonies were picked randomly per MacConkey agar plate and tested individually by PCR for *tsh*, *papC*, and *iucD* (no broth samples were positive for *cnf*). All isolates positive for any of these virulence genes were confirmed as *E*. *coli* by PCR detection of the *E*. *coli*-specific housekeeping gene *uidA* and underwent a more extensive virulence gene screen, as described below.

### Genotypic and phylogenetic screening

The 175 *E*. *coli* isolates identified by this initial screen as containing ≥ 1 of *tsh*, *papC*, and *iucD* were further screened by multiplex PCR for ExPEC status, based on detection of ≥ 2 of the following 5 ExPEC-defining traits: *papA* and/or *papC* (P fimbriae: counted as 1), *sfa/foc* (S and F1C fimbriae), *afa/dra* (Dr-binding adhesins), *kpsM* II (group 2 capsule), and *iutA* (aerobactin system) [26]. All isolates qualifying as ExPEC (n = 40), and a similar number of randomly selected non-ExPEC isolates (n = 37) from the remaining 135 *E*. *coli* isolates that did not qualify as ExPEC, underwent further analysis for major *E*. *coli* phylogenetic groups (A, B1, B2, and D) by triplex PCR [27] and were screened by multiplex PCR for 50 ExPEC-associated virulence genes (Table 1) [21, 28, 29].

**Table 1:** Prevalence of extraintestinal pathogenic *Escherichia coli* (ExPEC)-associated genes among chicken fecal *E*. *coli* isolates.

Functional category	Gene	No. of isolates positive^a^ (%)		*P*-value^b^
		ExPEC (n = 40)	Non-ExPEC (n = 37)	
Adhesin	F10	1 (3)	0 (0)	1.00
	F14	7 (18)	0 (0)	0.01
	*fimH*	36 (90)	36 (97)	0.36
	*hra*	15 (38)	9 (24)	0.23
	*iha*	1 (3)	1 (3)	1.00
	*papA*	11 (28)	0 (0)	< 0.001
	*papC*	14 (35)	0 (0)	< 0.001
	*papEF*	14 (35)	1 (3)	< 0.001
	*papG*2	38 (95)	1 (3)	< 0.001
	*pap*G3	39 (98)	0 (0)	< 0.001
	*sfa*	0 (0)	1 (3)	0.48
Protectin	*cvaC*	17 (43)	24 (65)	0.07
	*iss*	30 (75)	29 (78)	0.79
	*kpsMT*3	4 (10)	5 (14)	0.73
	*kpsMT* K1	2 (5)	0 (0)	0.49
	*kpsM II*	37 (93)	1 (3)	< 0.001
	*rfc*	0 (0)	1 (3)	0.48
	*traT*	0 (0)	1 (3)	0.48
Siderophore	*fyuA*	7 (18)	7 (19)	1.00
	*ireA*	8 (20)	11 (30)	0.43
	*iroN*	19 (48)	27 (73)	0.04
	*iutA*	40 (100)	35 (95)	0.23
Toxin	*astA*	13 (33)	11 (29)	0.81
	*hlyF*	36 (90)	37 (100)	0.12
	*pic*	2 (5)	1 (3)	1.00
	*tsh*	21 (53)	36 (97)	< 0.001
Miscellaneous	*ibeA*	1 (3)	0 (0)	1.00
	*malX*	3 (8)	0 (0)	0.24
	*ompT*	13 (33)	14 (38)	0.64
	*usp*	17 (43)	25 (68)	0.04

^a^All isolates were negative for the following adhesins (*afa/draBC*, *afaE*, *bmaE*, *clpG*, *focG*, F*11*, F*12*, F*16*, F*17*, *gafD*, *papG1*, *sfaS*), protectins (*kpsMT* K2, *kpsMT* K15, *kfiC* K5), toxins (*cdt*, *cnf1*, *hlyA*, *saT*), and *fliC* H7 gene.

^b^*P*-values determined by Fisher’s exact test, two-tailed.

### *In vitro* phenotypic screening

Study isolates underwent phenotypic screening for siderophore and colicin production, biofilm formation, complement resistance, growth in human urine, swimming motility, and cell association ability. Siderophore production was analyzed using Chrome azurol S agar as described previously [30]. A positive result consisted of bacterial colonies displaying orange haloes on blue agar after overnight incubation at 37°C; halo diameters were recorded. Total colicin production was tested using the double-agar diffusion method [31] on trypticase soy agar. *E*. *coli* K-12 χ6092 was used as a sensitive indicator for colicin production.

Biofilms were quantified in 96-well microtiter plates (Microtest^™^ U-Bottom, Becton Dickenson, Franklin Lakes, NJ) as described previously [32]. Bacterial strains were grown overnight to an optical density (OD) at 600 nm of 1.0, diluted 1:100 in PBS, and 200 μl of the culture was added to 96-well plates in quadruplicate. After overnight incubation at 37°C, plates were stained with crystal violet. Individual experiments were performed at least three times. A crystal violet-stained biofilm with an OD_600_ at least 3-fold greater than the negative control well containing only growth medium was considered a positive result.

Resistance to guinea pig serum complement was determined using a standard quantitative microtiter plate method [33]. Briefly, 10^4^ CFU of bacteria in 100 μl of PBS was mixed with an equal volume of 50% serum. After 4 h at 37°C, the OD_492_ was determined spectrophotometrically. Isolates were considered complement-resistant if the OD_492_ in serum-containing wells equaled or exceeded that of the no-serum control well. Heat-inactivated sera was used as a control.

Growth in human urine was assessed as described previously [32]. Urine was filter sterilized, pooled, and frozen in aliquots. Diluted bacterial suspensions in urine were prepared by adding to urine a 1:100 volume of an overnight LB culture after it had been adjusted to an OD_600_ of 1.0. The turbidity of the bacterial suspensions was measured using a wideband filter (420–580 nm) every 15 min for 8 h at 37°C. *E*. *coli* K-12 strain χ6092 was used as a negative control and UPEC strain CFT073 as a positive control.

For swimming motility assays, a toothpick was used to stab-inoculate overnight LB cultures adjusted to an OD_600_ of 1.0 onto 0.25% agar plates containing 0.7% sodium chloride and 1.3% tryptone. Plates were incubated for 8 h at 37°C.

T24 human bladder carcinoma (ATCC HTB-4) and A498 human renal carcinoma (ATCC HTB-44) cell lines were obtained from American Type Culture Collection (ATCC) and maintained in growth media as specified by ATCC. For inoculation onto cell monolayers, bacterial cultures were prepared from an LB overnight culture, diluted 1:100 in freshly pooled (4 individual samples) filter-sterilized human urine, and then incubated statically for 24 h at 37°C. Approximately 10^5^ CFU of bacteria were inoculated onto cells at a multiplicity of infection of 10. For bacterial association assays, the inoculated cells were incubated at 37°C in 5% CO_2_ for 1 h, then rinsed three times with PBS. Cells were lysed with 0.1% deoxycholic acid sodium salt for enumeration of viable colonies by serial dilution plating on MacConkey agar. For persistence assays, after the cells had been incubated with bacteria for 1 h and rinsed with PBS, medium containing 100 μg/ml gentamicin (Sigma-Aldrich) was added and cells were incubated at 37°C for an additional 1 or 3 h. Cells were then washed three times with PBS and lysed for serial dilution plating. Association was calculated as the ratio of the number of cell-associated bacteria at 1 h to the initial inoculum size, and persistence as the ratio of the number of intracellular bacteria at 3 h vs. 1 h.

### Virulence in chickens

Female white leghorn chickens (VALO BioMedia, Adel, IA) were raised on the floor in pens containing deep wood shavings to mimic cage-free conditions, and separate rooms were used for each bacterial challenge strain. Animals were maintained on a Purina^®^ non-medicated feed containing prebiotics and probiotics throughout the study. During the acclimation period prior to infection, 2 animals found with pecking wounds had died. At 5 weeks of age, chickens were inoculated with 10^7^ CFU via the air sac from an overnight LB culture suspended in PBS [17]. All experimental and control groups contained at least 7 animals. Chickens were monitored twice daily for 2 days and euthanized at 48 h post-infection by carbon dioxide inhalation. No chickens died following infection prior to the experimental endpoint. At 2 days post-challenge blood, heart, liver, lung, spleen, and an air sac swab were collected for detection and quantification of *E*. *coli* using MacConkey agar. Gross colibacillosis lesions in the air sac, heart, and liver were scored using an established scoring scheme [17].

### Virulence in mammals

Rodent models of human ExPEC infections, including sepsis, meningitis, and UTI, were used to evaluate the isolates' virulence potential for humans. Seven-week-old female BALB/c mice (Charles River Laboratories, Wilmington, MA) were injected intraperitoneally with approximately 10^8^ CFU of a log-phase LB culture suspended in PBS. All experimental and control groups contained 5 mice. Mice were observed daily over 7 days and scored for illness severity using an established scoring scheme [34] as follows: 1, healthy; 2, minimally ill; 3, moderately ill; 4, severely ill; 5, dead. All animals meeting endpoint criteria were euthanized by carbon dioxide inhalation, death was not considered an endpoint criterion. However, some animals died following infection prior to the experimental endpoint due to sepsis. On day 7, surviving mice were euthanized by carbon dioxide inhalation.

The ability of chicken fecal isolates to enter the central nervous system was tested in an established rat model of *E*. *coli* meningitis [35]. Briefly, outbred pregnant Sprague-Dawley rats (Charles River Laboratories) with timed conception were used to give birth to neonatal rats. Five-day-old Sprague-Dawley rats were divided randomly into groups of 10 to 12 rats and received approximately 10^2^ CFU intraperitoneally. No infected rats died prior to the experimental endpoint. At 18 h post-inoculation, rats were euthanized by carbon dioxide inhalation followed by cervical dislocation, and blood and cerebrospinal fluid specimens were collected, serially diluted, and plated on MacConkey agar.

The ability of bacteria to cause UTI was tested in mice, as described previously [36]. Seven to eight-week-old female CBA/J mice (Jackson Laboratories, Bar Harbor, ME) were inoculated via a urethral catheter with approximately 10^8^ CFU of bacteria. Mice were catheterized following anesthesia with an intraperitoneal injection of a ketamine—xylazine—acepromazine cocktail. Three isolates that grew in human urine (MM242, MM243, and MM244) and two that failed to grow (MM248 and MM259) were selected as experimental isolates. All experimental and control groups contained at least 9 mice. Mice were monitored twice daily for 2 days. No animals died following infection but prior to the experimental endpoint. Mice were euthanized 48 h post-infection by carbon dioxide inhalation and CFU/g of bladder, kidney, liver, and spleen were determined by serial dilution plating of organ homogenates on MacConkey agar.

### Statistical analysis

Fisher's exact test (two-tailed) was used to compare ExPEC and non-ExPEC isolates for the prevalence of ExPEC virulence genes and virulence-associated phenotypes, and experimental and control strains for the proportion of tissues positive for *E*. *coli* in the chicken colibacillosis model. A t-test was used to compare ExPEC and non-ExPEC isolates for colicin and siderophore production. An ANOVA followed by Dunnett’s method for multiple means comparison was used to compare experimental and control strains in cell association and persistence assays, and in the colibacillosis, meningitis, and UTI models. The Log-rank (Mantel-Cox) test was used to compare survival curves from the sepsis model. Analyses were performed using Graphpad Prism 6.0. *P* values < 0.05 were considered significant.

## Results

### Prevalence of ExPEC virulence genes

Fecal *E*. *coli* isolates (n = 304) from healthy chickens were prescreened for 4 genes (*tsh*, *papC*, *iucD*, and *cnf*) and 175 tested positive for one or more of these genes. Among the 175 isolates, 40 qualified as ExPEC using a PCR-based ExPEC screening method [37]. Extended virulence genotyping of the 40 ExPEC and 37 randomly selected non-ExPEC isolates identified 26 of the 50 genes investigated in at least one isolate each (Table 1 and S1 Table).

### Phylogenetic groups and subpathotypes

Prevalence of phylogroups differed for isolates classified as ExPEC vs. non-ExPEC for group A (63% vs. 8%, respectively: *P* < 0.001), B1 (0% vs. 78%: *P* < 0.001), and D (33% vs. 11%: *P* = 0.03), but not group B2 (5% vs. 3%: *P* = 1.0).

ExPEC isolates were classified into subpathotypes based on previously described criteria [32] (Table 2). Of the 40 ExPEC isolates, 32 (80%) qualified for one or more of the defined subpathotypes, including 24 (60%) as APEC (18% APEC only) and 15 (38%) as UPEC (18% UPEC only). In contrast, none qualified as NMEC, and 8 (20%) fit none of the defined pathotypes. Of the 32 APEC and UPEC isolates, 15 (47%) qualified additionally as SEPEC.

**Table 2:** Criteria and prevalence of extraintestinal pathogenic *Escherichia coli* (ExPEC) subpathotypes.

Subpathotype^a^	Selection-based criteria		No. (%)^c^
	Phenotype	Genotype^b^	
APEC	None	ExPEC and ≥ 4 of 5 selected APEC genes	7 (18)
NMEC	None	ExPEC plus *kpsMT* K1 and *ibeA*	0 (0)
UPEC	Growth in urine	ExPEC	7 (18)
Undefined	None	ExPEC	4 (10)
APEC/SEPEC	Complement resistant	ExPEC and ≥ 4 of 5 selected APEC genes	10 (25)
APEC/UPEC	Growth in urine	ExPEC and ≥ 4 of 5 selected APEC genes	3 (8)
APEC/UPEC/SEPEC	Growth in urine and complement resistant	ExPEC and ≥ 4 of 5 selected APEC genes	4 (10)
UPEC/SEPEC	Growth in urine and complement resistant	ExPEC	1 (3)
Undefined/SEPEC	Complement resistant	ExPEC	4 (10)

^a^APEC, avian pathogenic *E*. *coli*; NMEC, neonatal meningitis *E*. *coli*; SEPEC, sepsis-associated *E*. *coli*; Undefined, classified as ExPEC but does not correspond with any of the three major subpathotypes (APEC, NMEC, or UPEC); UPEC, uropathogenic *E*. *coli*.

^b^ExPEC defined by ≥ 2 of the following genes: *papA* and/or *papC* (counted as 1), *sfa/foc*, *afa/dra*, *kpsM* II, and *iutA*. For APEC, genes included: (1) *kpsM* II; (2) *iss*; (3) *tsh*; (4) one of the 5 genes: *sfa*, *foc*, *papA*, *papC*, and *papEF*; and (5) one of the 2 genes *iutA* and *fyuA*.

^c^The number of isolates positive for a given subpathotype only.

### *In vitro* virulence-associated phenotypes

PCR-confirmed ExPEC isolates and randomly selected non-ExPEC isolates were compared for virulence-associated phenotypes. For this, siderophore and colicin production, biofilm formation, complement resistance, and growth in human urine were assessed by standard assays (S2 Table). The results are summarized in Table 3.

**Table 3:** Prevalence of virulence-associated *in vitro* phenotypes among chicken fecal *Escherichia coli* isolates.

ExPEC^a^	No. of isolates	Siderophore production	Mean CAS^b^ zone diameter (mm)	Colicin production	Mean colicin zone diameter (mm)	Biofilm production	Complement resistance	Growth in urine
Yes	40	100%	18.4^c^	93%	18.5	100%^c^	48%	38%^c^
No	37	100%	14.4	92%	15.6	81%	24%	0%

^a^Extraintestinal pathogenic *E*. *coli*.

^b^Chrome azurol S (zone diameter indicates extent of siderophore production).

^c^Statistically significant difference, ExPEC vs. non-ExPEC (*P* < 0.05) determined by a t-test for mean zone diameter of colicin and siderophore production, and Fisher’s exact test (two tailed) for siderophore, colicin, and biofilm production, complement resistance, and growth in urine.

Selected isolates—chosen based on differing ExPEC status, genotype, phylogroup, and *in vitro* phenotypes—were additionally characterized for swimming motility, ability to associate with and persist within human A498 and T24 cells, and virulence in animal models of ExPEC-associated infections. These isolates were selected based on applicability to the animal challenge models. Thus, all APEC and two APEC/UPEC isolates were tested in chickens, two of three complement-resistant isolates were selected for the sepsis model, and APEC/UPEC isolates were selected for the UTI model. Since, no fecal *E*. *coli* isolates were classified as NMEC, isolates containing virulence factors (K1 capsule or *ibeA*) associated with NMEC were selected. In addition, non-ExPEC isolates were selected to determine if isolates not classified as ExPEC based on molecular typing could still cause ExPEC-associated diseases. Table 4 summarizes the isolates' relevant *in vitro* phenotypes. Since bladder and kidney cell lines were used for cell association and persistence assays, only isolates tested in the UTI mouse model were characterized in these assays.

**Table 4:** Characteristics of selected *Escherichia coli* isolates from chicken fecal samples used for *in vivo* experiments.

Isolate	ExPEC subpathotype or non-ExPEC	Phylo-group	Virulence genotype	CR	Urine growth	Siderophore	Colicin	Biofilm	Swim	A498 cells		T24 cells	
										A	P	A	P
MM149	APEC	B2	*astA*, *cvaC*, *fimH*, *fyuA*, *hlyF*, *ibeA*, *iroN*, *iss*, *iutA*, *kpsM* II, *malX*, *ompT*, *tsh*, *usp*	+	-	+	+	+	NS	NT	NT	NT	NT
MM218	APEC	A	*astA*, *fyuA*, *hlyF*, *hra*, *ire*, *iroN*, *iss*, *iutA*, K1, *kpsM* II, *tsh*	-	-	+	+	+	Sig-	NT	NT	NT	NT
MM225	Non-ExPEC	A	*hlyF*, *iss*, *ompT*, *tsh*	-	-	+	+	+	Sig-	NT	NT	NT	NT
MM242	APEC/UPEC	A	*cvaC*, *fimH*, *hlyF*, *iss*, *iutA*, *kpsM* II, *tsh*, *usp*	+	+	+	+	+	Sig-	Sig+	NS	Sig+	NS
MM243	APEC/UPEC	A	*cvaC*, *fimH*, *hlyF*, *iss*, *iutA*, *kpsM* II, *tsh*, *usp*	-	+	+	+	+	Sig-	NS	NS	NS	NS
MM244	APEC/UPEC	A	*cvaC*, *fimH*, *hlyF*, *iss*, *iutA*, *kpsM* II, *tsh*, *usp*	+	+	+	+	+	NS	NS	NS	NS	NS
MM248	Non-ExPEC	B1	*cvaC*, *fimH*, *hlyF*, *iroN*, *iss*, *iutA*, *tsh*, *usp*	-	-	+	+	-	Sig+	Sig+	NS	Sig+	Sig+
MM259	Non-ExPEC	B2	*fimH*, *hlyF*, *tsh*	-	-	+	+	+	Sig+	NS	NS	NS	Sig+
MM299	APEC	D	*astA*, *fimH*, *hlyF*, *iroN*, *iss*, *iutA*, *kpsM* II, *tsh*	+	-	+	+	+	Sig+	NT	NT	NT	NT

A, cell association assay; APEC, avian pathogenic *E*. *coli*; CR, complement resistance; ExPEC, extraintestinal pathogenic *E*. *coli*; P, cell persistence assay; Phylo, phylogenetic group; NS, not significantly (*P* < 0.05) different compared to negative control MG1655; NT, not tested; Sig+, significantly (*P* < 0.05) greater than negative control MG1655; Sig-, Significantly (*P* < 0.05) less than negative control MG1655; Swim, swimming motility; UPEC, uropathogenic *E*. *coli*.

### Ability to cause chicken airsacculitis

Using a chicken airsacculitis model, six chicken fecal *E*. *coli* isolates and positive controls APEC-O2 and χ7122 were compared with negative control MG1655 for invasion of the internal organs of chickens after inoculation via the air sac (Table 5). Isolates classified as APEC (MM149, MM218, and MM299), two of three APEC/UPEC (MM242 and MM243), and one non-ExPEC (MM259) were selected. Some test isolates and both positive controls, but not the negative control, yielded positive cultures for multiple internal organs. Bacterial counts for chicken fecal isolates exceeded those for negative control strain MG1655 in the spleen for isolates MM149 and MM299, and in the air sac and heart for isolate MM218.

**Table 5:** Ability of *Escherichia coli* isolates to cause systemic infection in chickens.

Strain	Mean lesion score		Air sac	Blood		Heart		Liver		Lung		Spleen	
	Air sac	Heart and liver	Pro-portion positive	Pro-portion positive	Mean log_10_ CFU/ml	Pro-portion positive	Mean log_10_ CFU/g	Pro-portion positive	Mean log_10_ CFU/g	Pro-portion positive	Mean log_10_ CFU/g	Pro-portion positive	Mean log_10_ CFU/g
**Controls**													
χ7122	2.1^a^	2.4^a^	8/10^a^	6/10	1.1 ± 1.2	9/10^a^	3.4 ± 1.6^a^	8/10^a^	1.9 ± 1.1^a^	7/10^a^	1.8 ± 1.3	9/10^a^	3.2 ± 1.3^a^
APEC-O2	1.0	1.6	3/8	1/8	0.6 ± 1.6	3/8	2.2 ± 3.2^a^	4/8	1.2 ± 1.4	4/10	1.8 ± 2.5	5/8^a^	2.1 ± 1.9^a^
MG1655	0.6	0.5	0/8	1/8	0.3 ± 0.7	0/8	0.0 ± 0.0	0/8	0.0 ± 0.0	0/8	0.0 ± 0.0	0/8	0.0 ± 0.0
**Fecal isolates**													
MM149	0.6	1.4	3/10	2/10	0.8 ± 1.6	2/10	0.5 ± 1.1	2/10	0.8 ± 1.8	4/10	1.2 ± 1.6	5/10^a^	1.5 ± 1.8
MM218	1.0	1.9	4/7^a^	1/7	0.5 ± 1.2	4/7^a^	2.2 ± 2.3^a^	3/7	1.3 ± 1.8	3/7	1.7 ± 2.1	3/7	1.4 ± 1.9
MM242	0.0	0.4	3/8	0/8	0.0 ± 0.0	1/8	0.2 ± 0.5	1/8	0.2 ± 0.7	1/8	0.4 ± 1.2	1/8	0.4 ± 1.2
MM243	0.1	0.6	0/7	1/7	0.4 ± 1.0	1/7	0.3 ± 0.7	1/7	0.2 ± 0.6	1/7	0.4 ± 0.9	1/7	0.4 ± 1.1
MM259	0.3	0.1	1/8	1/8	0.6 ± 1.7	1/8	0.3 ± 0.7	1/8	0.4 ± 1.1	1/8	0.5 ± 1.4	2/8	0.7 ± 1.5
MM299	0.3	0.9	3/10	5/10	1.0 ± 1.3	1/10	0.3 ± 0.8	3/10	0.8 ± 1.3	4/10	1.4 ± 2.0	6/10^a^	1.8 ± 1.7

Concentration data is represented by mean values ± standard deviation. Counts were determined at 48 h post-inoculation

^a^Significant difference (*P* < 0.05) compared with MG1655 (negative control) determined by a Fisher’s exact test (two tailed) for the proportion positive, or by an ANOVA followed by Dunnett’s method for mean bacterial loads.

### Ability to cause sepsis

**Fig 1:**
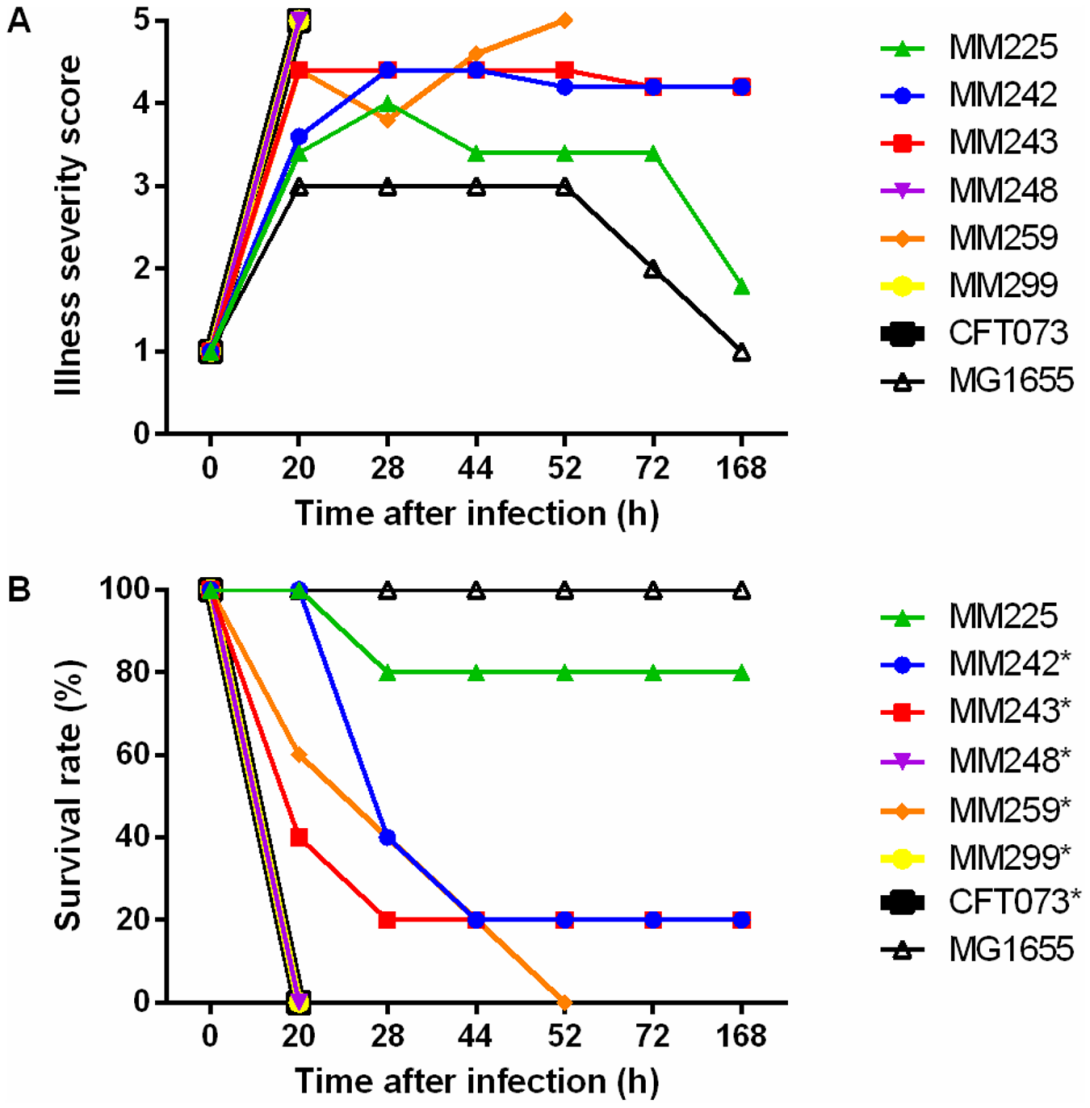
Ability of fecal *Escherichia coli* isolates to cause lethal sepsis in mice. A BALB/c mouse sepsis model was used to evaluate the ability of *E*. *coli* isolates to cause lethal sepsis within 7 days of intraperitoneal challenge with 10^8^ CFU. Five mice were used per strain. (A) Severity scores, as recorded over the week using a 5-point scoring scheme (1, healthy; 2, minimally ill; 3, moderately ill; 4, severely ill; 5, dead). (B) Survival rate over 7 d. Human ExPEC isolate CFT073 was used as a positive control and *E*. *coli* K-12 MG1655 as a negative control. An asterisk (*) represents a significantly (*P* < 0.05) different survival curve determined by The Log-rank (Mantel-Cox) test for experimental isolates or positive control strain CFT073 compared with the negative control MG1655.

Using a mouse sepsis model, three chicken fecal isolates classified as ExPEC (MM242, MM243, and MM299) and three as non-ExPEC (MM225, MM248, and MM259) were randomly selected, and along with positive control CFT073 were compared with negative control MG1655 for illness severity score and survival (Fig 1). During the experiment, 29 of 40 mice died from sepsis infection or were euthanized due to meeting endpoint criteria. Survival curves (Fig 1) were significantly different from negative control strain MG1655 for positive control strain CFT073 (*P* = 0.003) and fecal ExPEC isolates MM242 (*P* = 0.01), MM243 (*P* = 0.01), and MM299 (*P* = 0.003), and non-ExPEC isolates MM248 (*P* = 0.003) and MM259 (*P* = 0.002). In contrast, the non-ExPEC isolate MM225 was lethal in only one of five mice and the survival curve was not significantly different (*P* = 0.3) from the negative control.

### Ability to cause meningitis

**Fig 2:**
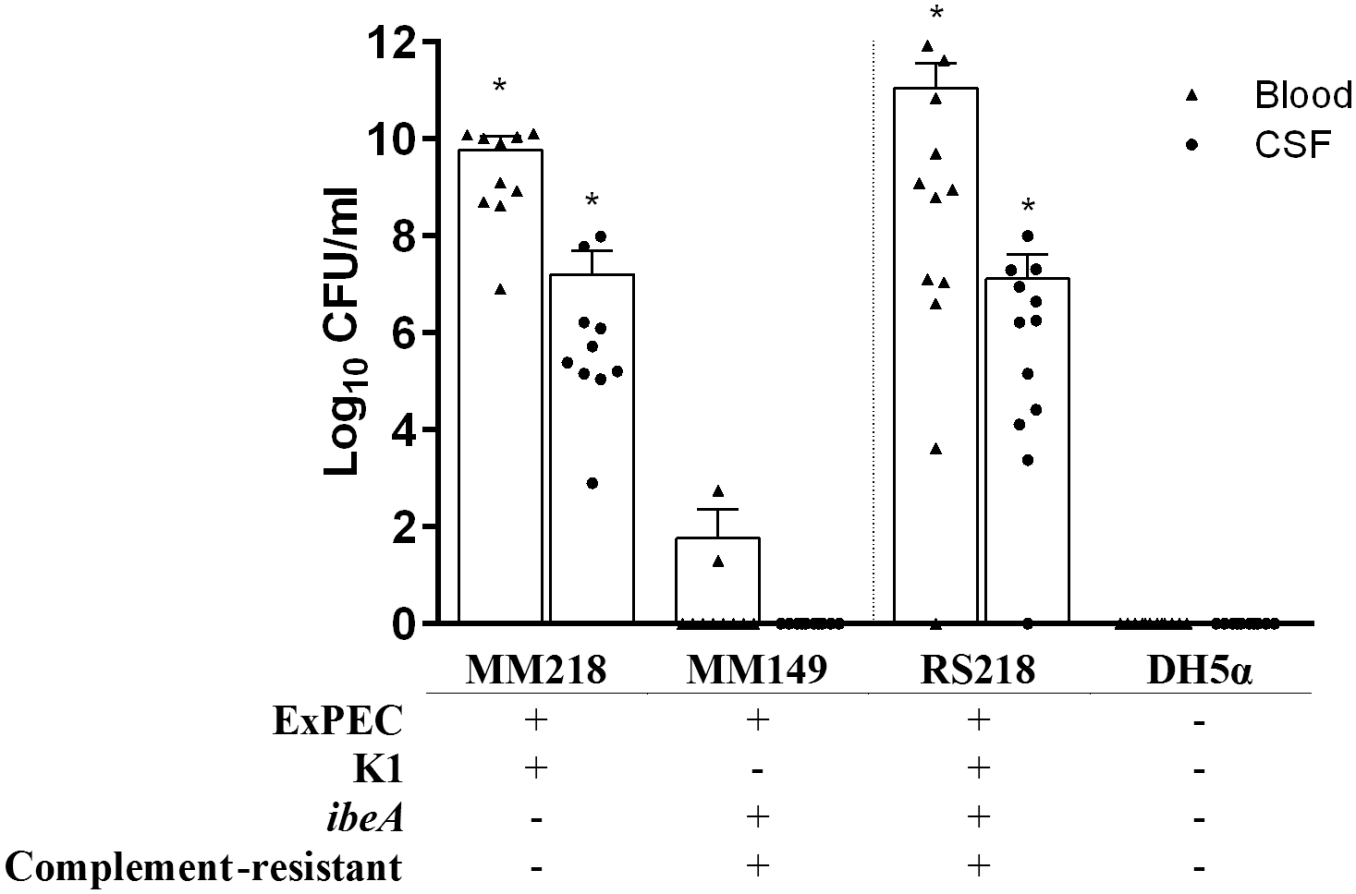
Ability of fecal *Escherichia coli* isolates to cause meningitis in rats. *E*. *coli* MM149 and MM218 isolated from chicken feces, positive control neonatal meningitis strain RS218, and negative control strain DH5α were assessed for their abilities to induce septicemia and meningitis in 5 day-old Sprague-Dawley rats. Isolates were characterized for extraintestinal pathogenic *E*. *coli* (ExPEC) status, K1 capsule, meningitis-associated gene *ibeA*, and complement resistance. Rats were challenged intraperitoneally with 10^2^ CFU and assessed 18 h later for bacterial concentration in blood (triangles) and cerebral spinal fluid (CSF) (circles). Each experimental group contained at least 10 rats. Each symbol represents an individual animal and the vertical dashed line separates chicken fecal *E*. *coli* isolates from control strains. An asterisk (*) represents significantly (*P* < 0.05) higher mean values determined by an ANOVA followed by Dunnett’s method for experimental isolates or positive control strain RS218 compared with the negative control DH5α.

Since no chicken fecal *E*. *coli* isolates were classified as NMEC based on molecular identification of both K1 and *ibeA*, isolates that were positive for K1 or *ibeA* were selected to be tested in the rat meningitis model. Chicken fecal isolates MM149 and MM218, which qualify as ExPEC but differ for complement resistance, K1 capsule, and the NMEC-associated invasin gene *ibeA* (Fig 2), were tested for their ability to cause meningitis in a neonatal rat model in comparison with human NMEC isolate RS218 and negative control DH5α. MM218 was recovered from the blood and cerebral spinal fluid at a similar level to NMEC isolate RS218, and at a significantly higher level compared with negative control DH5α. In contrast, MM149 was recovered inconsistently from blood (< 10^2^ CFU/ml) and not at all from CSF, and for neither endpoint differed significantly from negative control DH5α (Fig 2).

### Ability to cause urinary tract infection

**Fig 3:**
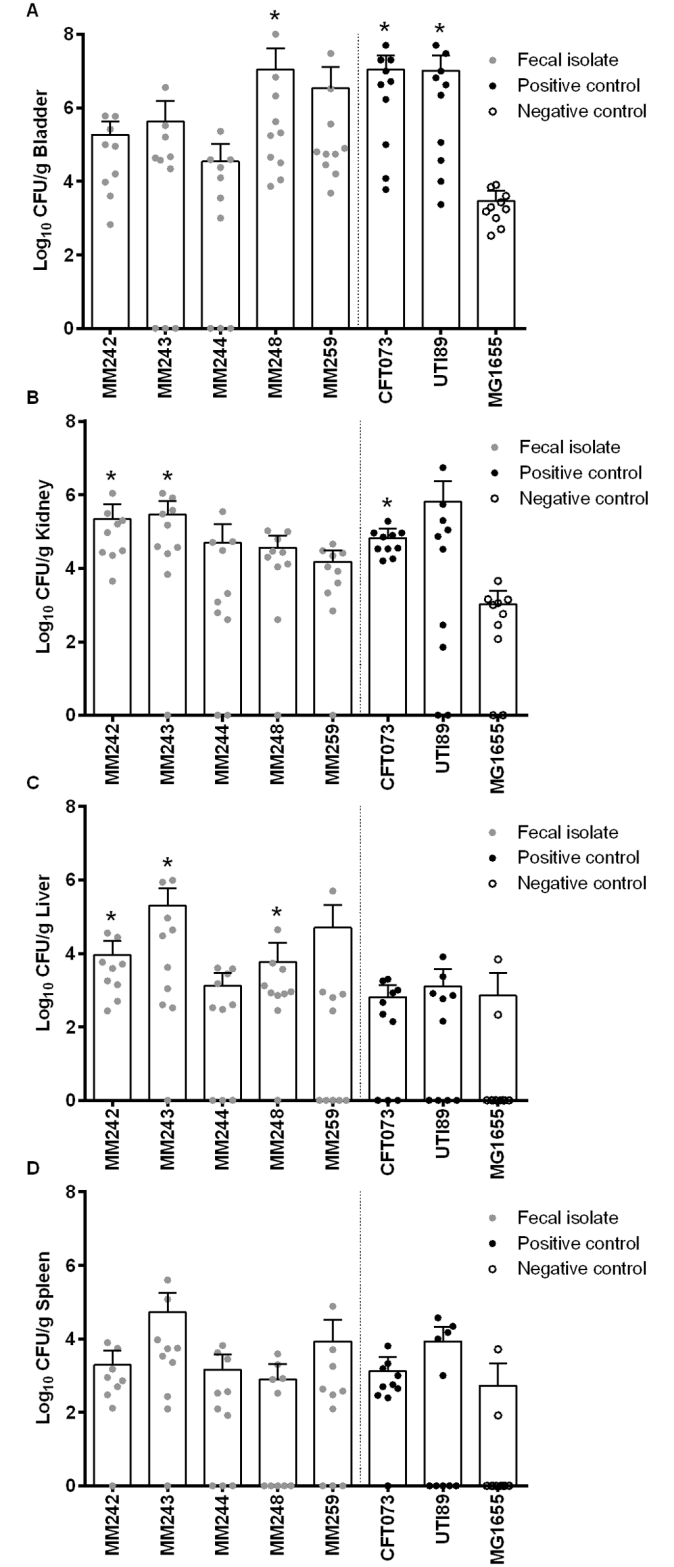
Ability of fecal *Escherichia coli* isolates to urinary tract infection. *E*. *coli* isolates MM242, MM243, MM244, MM248, and MM259, positive controls CFT073 and UTI89, and negative control MG1655 were assessed for their ability to colonize the (A) bladder and (B) kidney, and to invade in the (C) liver and (D) spleen of CBA/J mice. Mice were challenged with 10^8^ CFU via a urethral catheter and monitored for 2 days. Each experimental group contained at least 9 mice. Each dot represents an individual animal; the vertical dashed line separates chicken fecal *E*. *coli* isolates from control strains. An asterisk (*) represents significantly higher mean values determined by an ANOVA followed by Dunnett’s method for fecal *E*. *coli* isolates and positive control strains compared with the negative control.

In the mouse model of ascending UTI, bacterial loads were quantified in the bladder, kidney, liver, and spleen of mice 48 h after inoculation of 10^8^ CFU of the challenge strain into the bladder (Fig 3). Of the fecal *E*. *coli* isolates, three ExPEC isolates (MM242, MM243, and MM244) that could grow in urine and two non-ExPEC (MM248 and MM259) that failed to grow were selected. Some chicken fecal *E*. *coli* isolates equalled or exceeded one or both positive controls for bacterial counts in the internal organs. Significantly greater bacterial loads than observed with negative control MG1655 were observed in the bladder for both positive control strains and for the non-ExPEC fecal isolate MM248; in the kidney for positive control strain CFT073 and for APEC/UPEC fecal isolates MM242 and MM243; in the liver for fecal isolates MM242, MM243, and MM248; and in the spleen no significant differences were observed.

## Discussion

The presence and characteristics of pathogenic *E*. *coli* colonizing healthy production chickens could be important to both animal and human health. Here, we characterized *E*. *coli* isolates from the feces of healthy production chickens both genotypically and phenotypically, including for their ability to cause disease in animal models of chicken and human infections. Based on the molecular criteria of Johnson et al. [37], 13% (40/304) of the present chicken fecal *E*. *coli* isolates qualified as ExPEC. Varying isolation methods, classification methods, geographic locations, and management practices likely contribute to differences in frequency of ExPEC isolation between studies. In a previous study using methods different than that of the current study, 10% of *E*. *coli* isolates from feces of commercial egg layer and meat chickens qualified molecularly as ExPEC [38]. These findings indicate that commercial chickens can harbor *E*. *coli* isolates with virulence characteristics of ExPEC that could be transmitted to other chickens in the production house or contaminate carcasses during processing. Notably, one study recovered *E*. *coli* from 87% (691/798) of post-chill chicken carcasses at large commercial harvest facilities [39]. Although concentrations decreased with subsequent processing steps, low counts persisted, suggesting the possibility of contaminated retail poultry products, as documented in multiple retail market surveys [32, 40].

We analyzed for major *E*. *coli* phylogenetic groups to further characterize the virulence potential of the present study isolates. As found previously for isolates from chicken meat and eggs [32], phylogroup distribution varied in relation to ExPEC status, with most ExPEC isolates representing phylogroups A and D, and non-ExPEC isolates phylogroup B1. This is consistent with the fact that APEC strains belong predominantly to phylogroups A and D, whereas human-source ExPEC strains belong mainly to phylogroups B2 and D [13, 41, 42].

Genotypic tests that distinguish ExPEC from non-ExPEC isolates have been proposed [21, 37, 43–45], and have been used in previous studies to predict the zoonotic potential of animal-source ExPEC isolates [10, 13, 21]. However, certain *in vitro* phenotypes (e.g., biofilm formation, colicin production, complement resistance) for which straightforward genetic screens are unavailable also contribute to, or correspond with, the ability of *E*. *coli* to cause extraintestinal infections. We showed previously a correlation between complement resistance and the ability of APEC to invade the internal organs of experimentally challenged chickens [46]. Here, we found that virulence-associated *in vitro* phenotypes were more prevalent among ExPEC isolates than non-ExPEC isolates, and significantly so for biofilm formation and growth in urine. Biofilm formation, which has been identified as important for UPEC colonization [47], provides bacteria with protection from detergents, antibiotics, and host defense elements [48]. Here, we detected biofilm formation for all ExPEC isolates. Analogous to the contribution of biofilm to bacterial persistence in the genitourinary tract [49], biofilm formation may also allow bacteria to persist on surfaces of poultry products such as raw meat and eggs, a suitable topic for future study.

To survive in extraintestinal sites, bacteria must not only overcome harsh conditions but also acquire nutrients, including micronutrients such as iron. Iron acquisition is critical in the iron-limited environment of the urinary tract. Most *E*. *coli* produce the catecholate siderophore enterobactin [50], possibly explaining why most of the present study isolates exhibited siderophore production. However, the ExPEC isolates produced larger haloes in that assay than did non-ExPEC isolates, suggesting that ExPEC produce more enterobactin and/or additional siderophores, e.g., aerobactin, salmochelin, and yersiniabactin [51, 52]. Whether siderophore production also contributes to bacterial persistence and survival on raw poultry meat and eggs warrants study.

Animal models of poultry and human infections were used to assess the ability of the study isolates to cause ExPEC-associated infections. To our knowledge, this is the first study to test *E*. *coli* from the feces of healthy production chickens in four ExPEC disease models, including avian colibacillosis, sepsis, meningitis, and UTI. In the avian colibacillosis model, chicken-source *E*. *coli* isolates were recovered from multiple internal organs, supporting that initially uncolonized chickens in production houses could acquire potentially invasive *E*. *coli* that are shed by colonized birds. Our findings confirm those of a previous study in which *E*. *coli* isolates from chicken feces and the poultry house environment invaded the internal organs of challenged chickens [7].

In previous studies of poultry-source *E*. *coli* in rodent models of ExPEC-associated human infections, Johnson et al. found that an APEC turkey lung isolate lacked full virulence in a mouse sepsis model and failed to cause bacteremia or meningitis in a rat meningitis model [53], whereas Tivendale et al. found that some avian colibacillosis isolates caused bacteremia and meningitis in the rat meningitis model [54]. Production chickens with colibacillosis may die because of infection or have their carcasses condemned when the lesions are identified during processing [3], which could reduce the risk of transfer of APEC strains from infected chickens to meat products during processing. However, if ExPEC are harbored in chicken feces, they may pose a less apparent but nonetheless real risk to food safety. To our knowledge, the present study is the first to show that a fecal *E*. *coli* isolate (MM218) from a healthy production chicken can cause bacteremia and meningitis in a rat meningitis model. The same isolate invaded internal organs of chickens demonstrating the potential of *E*. *coli* from chickens to cause disease in both poultry and humans. In addition, some of the studied fecal isolates caused lethal sepsis in ≤ 20 h, similar to human ExPEC strain CFT073. Fecal isolate MM299 caused lethal sepsis ≤ 20 h and invaded in the spleen of challenged chickens at significant levels, further demonstrating the potential of *E*. *coli* from chickens to cause disease in both poultry and humans. These findings have potentially important implications for food safety, since they suggest that chickens without colibacillosis could, via fecal contamination at harvest, transfer to poultry meat *E*. *coli* isolates with the ability to cause human meningitis and sepsis.

In the United States, UTI-related healthcare costs exceed $1 billion per year [6], and food-producing animals have been identified as a potential source of human ExPEC infection [11]. We found that 19% (15/77) of the tested chicken fecal *E*. *coli* isolates had the potential to cause UTI, based on their *in vitro* growth in urine, and that these isolates were mostly ExPEC per molecular criteria. These findings support a previous study that identified 23% of ExPEC isolates from raw chicken meat as UPEC [32]. We found that some chicken-source isolates could cause UTI in a mouse model, and with similar intensity as observed for positive control strains from humans with cystitis and pyelonephritis. This confirms in a very different geographical region the findings of previous studies that *E*. *coli* isolates from the feces of healthy Danish broiler chickens were virulent in the UTI mouse model [41, 55]. To further implicate bacterial isolates from chickens as a cause of disease in humans, another study [56] demonstrated nearly identical pulsed-field gel electrophoresis profiles between isolates from chickens and humans. However, additional studies are needed to establish that ExPEC are transferred from animals to humans via contaminated meat and to define the frequency of such transfer.

Some fecal isolates that caused diseases in animal models tested were classified as non-ExPEC by the ExPEC-genotypic and phenotypic associated criteria. Future characterization such as genomic and high-throughput sequencing of these isolates could elucidate their mechanisms of virulence and improve ExPEC detection criteria.

## Conclusions

Our study provides an in-depth assessment of virulence-related genotypes and phenotypes, including *in vivo* virulence, of fecal ExPEC isolates from healthy production chickens. Multiple methods were used to identify isolates with presumptive zoonotic potential. Some isolates were able to cause one or several diseases in animal models of septicemia, meningitis, UTI, and avian colibacillosis. Thus, this study provides the strongest evidence to date that chicken feces could be a source of virulent ExPEC that are able to infect humans and poultry. Interventions that reduce these pathogens in the chicken intestine and on carcasses and meat products could help to reduce transmission via poultry products and thus prevent clinical ExPEC infections and humans.

## Supporting information

S1 Table Virulence-associated genes analyzed. (XLSX)

S2 Table Virulence-associated phenotypes analyzed. (XLSX)

S3 Table Sepsis lethality and illness severity in mice. (XLSX)

S4 Table Bacterial load enumerated from blood and cerebral spinal fluid of infected animals. (XLSX)

S5 Table Bacterial load enumerated from different organs in intraurethrally challenged mice. (XLSX)
